# Development and validation of a model for predicting the risk of cardiovascular events in maintenance hemodialysis patients

**DOI:** 10.1038/s41598-024-55161-y

**Published:** 2024-03-21

**Authors:** Meijie Qin, Yuqi Yang, Lu Dai, Jie Ding, Yan Zha, Jing Yuan

**Affiliations:** 1 Zunyi Medical University, Guizhou, 563003 China; 2 Department of Nephrology, Guizhou Provincial People’s Hospital, Guizhou, 550002 China; 3 Department of Nephrology, The Second Affiliated Hospital of Guizhou University of Traditional Chinese Medicine, Guizhou, 550002 China; 4 The Second Clinical Medical College of Guizhou University of Traditional Chinese Medicine, Guizhou, 550002 China

**Keywords:** Cardiology, Medical research, Nephrology

## Abstract

The mortality rates for patients undergoing hemodialysis (HD) remain unacceptably high compared to the general population, and more specific information about the causes of death is not known. The study aimed to develop and validate a risk prediction model that uses common clinical factors to predict the probability of cardiovascular events in maintenance hemodialysis (MHD) patients. The study involved 3488 adult patients who received regular scheduled hemodialysis treatment at 20 hemodialysis centers in southwest China between June 2015 and August 2020, with follow-up until August 2021. The optimal parameter set was identified by multivariable Cox regression analyses and Cross-LASSO regression analyses and was used to establish a nomogram for predicting the risk of cardiovascular events in maintenance hemodialysis patients at 3 and 5 years. The performance of the model was evaluated using the consistency index (Harrell’s C-index), the area under the receiver operating characteristic (ROC) curve, and calibration plots. The model was validated by tenfold cross-validation and bootstrapping with 1000 resamples. In the derivation cohort, the model yields an AUC of 0.764 [95% confidence interval (CI), 0.737–0.790] and 0.793 [CI, 0.757–0.829] for predicting the risk of cardiovascular events of MHD patients at 3 and 5 years. In the internal validation cohort AUC of 0.803 [95% CI, 0.756–0.849], AUC of 0.766 [95% CI, 0.686–0.846], and the external validation cohort AUC of 0.826 [95% CI, 0.765–0.888], AUC of 0.817 [95% CI, 0.745–0.889] at 3 and 5 years. The model’s calibration curve is close to the ideal diagonal. By tenfold cross-validation analyses, the 3- and 5-year risk of cardiovascular events (AUC 0.732 and 0.771, respectively). By the bootstrap resampling method, the derivation cohort and validation cohort (Harrell’s C-index 0.695 and 0.667, respectively) showed good uniformity with the model. The constructed model accurately predicted cardiovascular events of MHD patients in the 3rd and 5th years after dialysis. And the further research is needed to determine whether use of the risk prediction tool improves clinical outcomes.

## Introduction

Chronic Kidney Disease (CKD) is a progressive disease that affects > 10% of the general population worldwide, totaling more than 800 million people^1^. Until 2021, the incidence of hemodialysis in China was 519.3 pmp. Among these, over 749,000 were maintenance hemodialysis (MHD) patients. The number of patients who died increased from 13,861 in 2011 to 57,873 in 2021. CKD is considered an accelerator and independent risk factor of cardiovascular disease^2^. The study analyses^3^ showed that cardiovascular events, cerebrovascular events, and infections accounted for 40%, 35.9%, and 9.9%, respectively, of the causes of death in hemodialysis patients in China.

The international kidney guidelines^4,5^ recommend early detection and prevention of cardiovascular events among MHD patients. They have small sizes, single-center and cross-sectional studies, and non-cardiovascular events with MHD, even though these clinical prediction models in existing can effectively predict the death risk of HD patients to a certain. Herein, we aimed at developing and validating an easy-to-use model for predicting the risk of the 3- and 5-year cardiovascular events of patients undergoing MHD. We hope it can help clinicians formulate individualized treatment and management strategies for CKD patients, reduce the risk of death, and prevent clinically important complications.

## Results

Table 1 describes event rates and characteristics for patients included in the derivation and validation sets. A total of 1588 (56.5%) patients had a first cardiovascular event, and 1224 (43.5%) patients had no cardiovascular event (eTable 1 in the Supplement). It was randomly divided into a derivation, internal and external validation set in a ratio of 7:2:1. In the derivation set of 1969 patients (mean [SD] age, 56 (45–67) years; 1174 [59.6%] males; 795 [40.4%] females). In the internal validation set of 562 patients (mean [SD] age, 56 (45–67) years; 341 [60.7%] males; 221 [39.3%] females). And in the external validation set of 281 patients (mean [SD] age, 56 (47–66) years; 179 [63.7%] males; 102 [36.3%] females) (Table 1). There is no difference between the derivation and the validation set (*P* > 0.05).

**Table 1:** Study population characteristics.

Characteristic	Derivation (N = 1969)	Internal validation^Δ^ (N = 562)	External validation^Δ^ (N = 281)
Cardiovascular event (yes/no)	1137:832	288:274	163:118^Δ^
Sex (male/female)	1174:795	341:221	179:102
Age, mean (SD), year	56 (45–67)	56 (44–67)	56 (47–66)
Dialysis mode (HD/HD + HP; HDF)	1319:650	364:198	187:94
Intradialytic hypotension (yes/no)	434:1535	125:437	61:220
Intradialytic hypertension (yes/no)	650:1319	178:384	83:198
BMI (kg/m^2^)	22.80 (20.57–25.15)	22.65 (20.29–25.05)	22.80 (20.64–25.54)
WHR (cm)	0.93 (0.87–0.97)	0.93 (0.88–0.97)	0.94 (0.89–0.98)
Handgrip (kg)	19 (14–26)	19 (14–26)	21 (15–26)
ECW	14.20 (12.30–16.30)	14.10 (12.40–16.10)	14.50 (12.60–16.40)
LTI	14.10 (12.40–16.30)	14.00 (12.30–16.18)	14.40 (12.40–16.60)
FAT	15.10 (9.90–20.90)	14.80 (9.50–20.70)	15.20 (10.00–20.70)
BAS (%)	0.50 (0.30–0.70)	0.50 (0.30–0.70)	0.50 (0.40–0.80)
LYMPH (10^9^/L)	1.09 (0.85–1.41)	1.09 (0.80–1.37)	1.10 (0.86–1.40)
Platelet distribution density	15.50 (11.90–16.50)	15.80 (12.20–16.57)	15.80 (12.30–16.70)
TB (μmol/L)	7.20 (5.20–10.06)	7.09 (5.33–9.71)	7.35 (5.50–10.00)
DB (μmol/L)	2.39 (1.70–3.30)	2.40 (1.63–3.50)	2.39 (1.60–3.47)
Dialysis vintage (months)	49 (29–78)	50 (29–85)	46 (26–86)
Dialysis frequency (/week)			
1	20 (1.0%)	5 (0.9%)	2 (0.7%)
2	318 (16.2%)	104 (18.6%)	36 (12.8%)
3	1624 (82.5%)	448 (79.8%)	242 (86.1%)
4	7 (0.3%)	4 (0.7%)	1 (0.4%)
Hypertension (yes/no)	1712:257	474:88	252:29
Diabetes (yes/no)	585:1384	169:393	87:194

*BMI* body mass index, *BAS* basophil ratio, *DB* direct bilirubin, *ECW* extracellular water, *FAT* fat content, *HD* hemodialysis, *HP* hemoperfusion, *HDF* hemodiafiltration, *LTI* lean tissue index, *LYMPH* number of lymphocytes, *TB* total bilirubin, *WHR* waist-hip ratio; ^Δ^*P* > 0.05.

### Prediction of cardiovascular events in MHD patients in the derivation cohort

**Figure 1:**
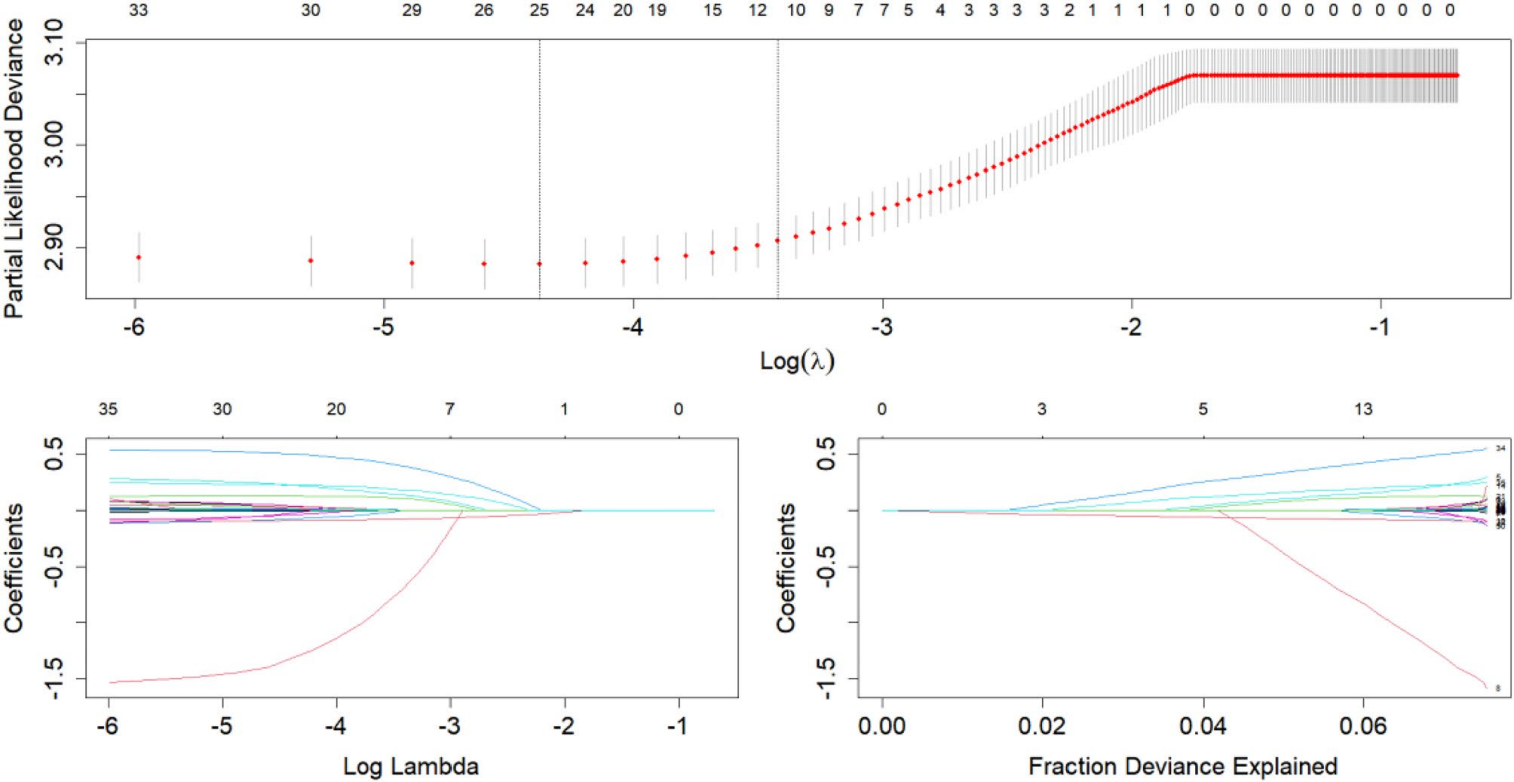
Cross-sectional LASSO regression analysis. The lambda min (λ minimum) selected 26 variables and the lambda 1-SE (λ-consistent selection) reduced the 37 variables to 12.

All variables have been analyzed for correlation, excluding highly correlated indicators (Variance inflation factor test, VIF < 10). To control the interaction between multiple predictor factors and reduce the overfitting, the optimal model was finally determined by constructing multiple models for comparison. First, univariate survival Cox regression analyses were performed using R software to elect 17 predictors (eTable 2 in the Supplement). Before the multivariate survival Cox regression analyses, PH assumption analyses were performed through the *Cox. zph* function (*P* > 0.05), and the variables that according to the PH assumption were subjected to multivariate survival Cox regression analyses, and the final predictors were determined to be age, mode of dialysis, whether or not intradialytic hypotension, waist-to-hip ratio, handgrip strength, lean tissue index, lymphocyte, platelet distribution density, total bilirubin, age on dialysis, and combined with hypertension and/or diabetes mellitus, and was named Model 1 (eTable 3 in the Supplement). Cross-LASSO regression analyses of survival data were performed using the glmnet R package. The results showed (Fig. 1) that lambda min (λ minimum) selected 26 variables, and those that conformed to the PH assumptions were subjected to multivariate survival Cox regression analyses, and finally obtained 13 predictors, named Model 2 (eTable 4 in the Supplement). The lambda 1-SE (λ-consistent selection) reduced 37 variables to 12 (Fig. 1), which were age, mode of dialysis, whether or not intradialytic hypotension, waist-to-hip ratio, handgrip strength, lean tissue index, monocyte, platelet distribution density, total bilirubin, age on dialysis, and combined with hypertension and/or diabetes mellitus was named Model 3.

The Harrell’s C-indexes for Models 1, 2, and 3 were 0.680, 0.681, and 0.680. The three models were compared separately using the *compareC* R package. The *Z* value of model 1 and model 2 is − 0.968, and the *P* value is 0.333, indicating that there is no statistical difference between the two models. The difference between model 1 and model 3 was − 0.001, *Z* value of − 0.114, and the *P* value of 0.909, with no significant difference between the two models (Table 2). Therefore, model 2 was selected as the optimal set for the development and validation of a model for predicting the 3- and 5-year cardiovascular events in MHD, which predictors including age, mode of dialysis, whether or not intradialytic hypotension, waist-to-hip ratio, handgrip strength, extracellular water, lean tissue index, lymphocyte, platelet distribution density, total bilirubin, age on dialysis, and combined with hypertension and diabetes mellitus.

**Table 2:** Comparison between the three models.

*P*-value	Model 1 (*P*-value)	Model 2(*P*-value)	Harrell’s C-index
Model 1	–	0.333	0.680 (0.661–0.698)
Model 2	0.333	–	0.681 (0.662–0.699)
Model 3	0.909	0.674	0.680 (0.661–0.699)

The *P*-value for both Model 1 and Model 2 is 0.333 and for Model 3 is 0.909. The Harrell’s C-index for Model 1 is 0.680 [95% CI 0.661–0.698], for Model 2 is 0.681 [95% CI 0.662–0.699], and for Model 3 is 0.680 [95% CI 0.661–0.699].

**Figure 2:**
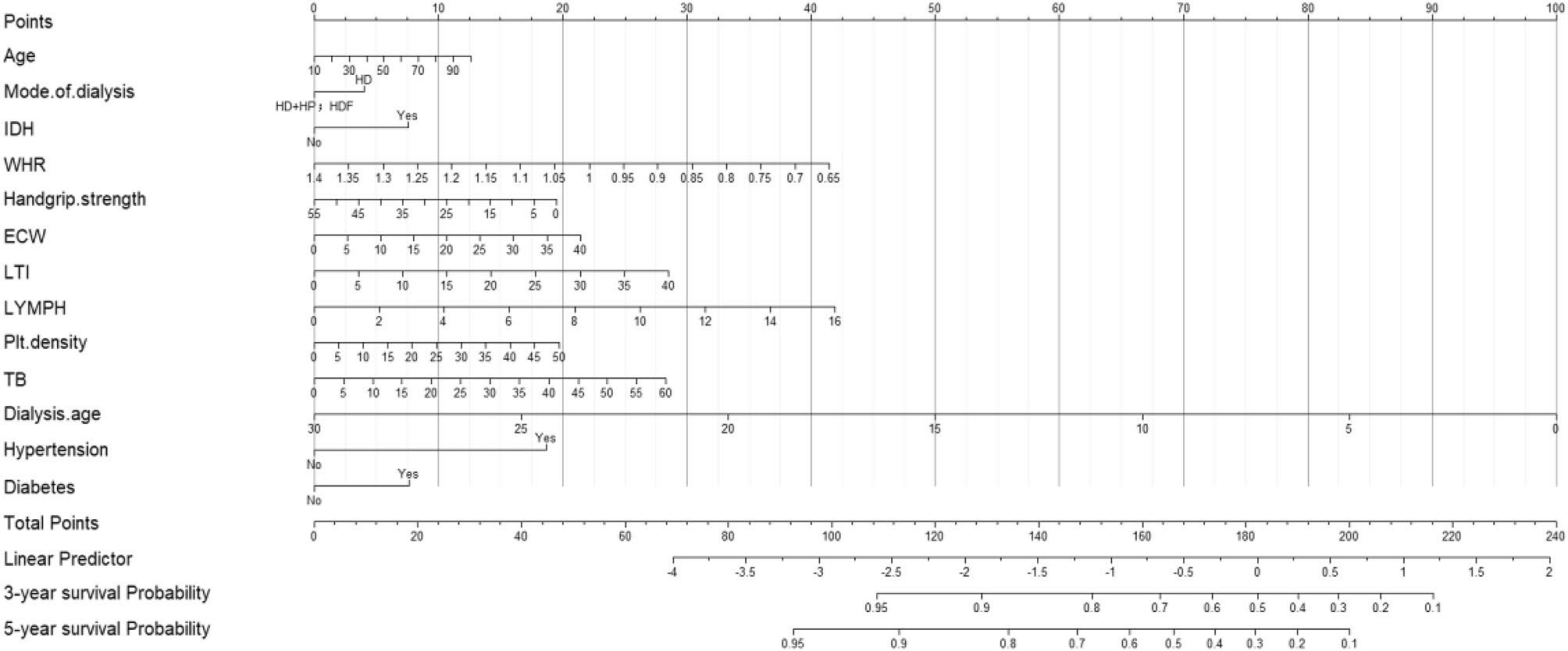
Nomogram for predicting the risk of cardiovascular events in maintenance hemodialysis patients. The length of each variable axis in the nomogram varies. First, draw a line upward to determine the fraction of each variable in the point axis. Second, find the location of the summed scores of these variables on the total score axis. Finally, a line was drawn on the morbidity axis and the values were summed up to determine the likelihood of a patient's risk of cardiovascular events at years 3 and 5 of hemodialysis. *ECW* extracellular water, *IDH* intradialytic hypotension, *LTI* lean tissue index, *LYMPH* number of lymphocytes, *TB* total bilirubin, *WHR* waist-hip ratio.

The Nomogram was developed from the risk proportions after transforming the coefficients of each independent variable to predict the risk of cardiovascular events in long-term hemodialysis patients (Fig. 2). Each variable corresponded to a score (from 0–100) and a total score (from 0–240). In addition, the total risk score was converted to predict the risk of cardiovascular events at 3 and 5 years.

### Model performance in validation cohort

**Figure 3:**
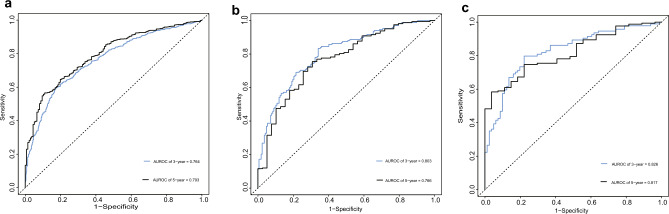
Derivation and validation set of 3 and 5-year ROC curves. (**a**) Time-dependent receiver-operating characteristic (ROC) curves of the derivation set cardiovascular event risk prediction in 3 (blue), and 5 (black) years. (**b**) Time-dependent receiver-operating characteristic (ROC) curves of the internal validation set cardiovascular event risk prediction in 3 (blue), and 5 (black) years. (**c**) Time-dependent receiver-operating characteristic (ROC) curves of the external validation set cardiovascular event risk prediction in 3 (blue), and 5 (black) years.

The Harrell’s c-index of the model was 0.681 [95% CI, 0.662–0.699], the internal validation set was 0.718 [95% CI, 0.684–0.751], and the external validation set was 0.742 [95% CI, 0.698–0.784]. The ROC for predicting the risk of cardiovascular events in MHDs at years 3 and 5 was 0.764 [95% CI, 0.737–0.790] and 0.793 [95% CI, 0.757–0.829] (Fig. 3a). The ROC for the risk of cardiovascular events at years 3 and 5 of the internal validation set was 0.803 [95% CI, 0.756–0.849] and 0.766 [95% CI, 0.684–0.751] (Fig. 3b), and the external validation set was 0.826 [95% CI, 0.765–0.888] and 0.817 [95% CI, 0.745–0.889] (Fig. 3c). As expected, our model performed well in predicting the occurrence of cardiovascular events.

**Figure 4:**
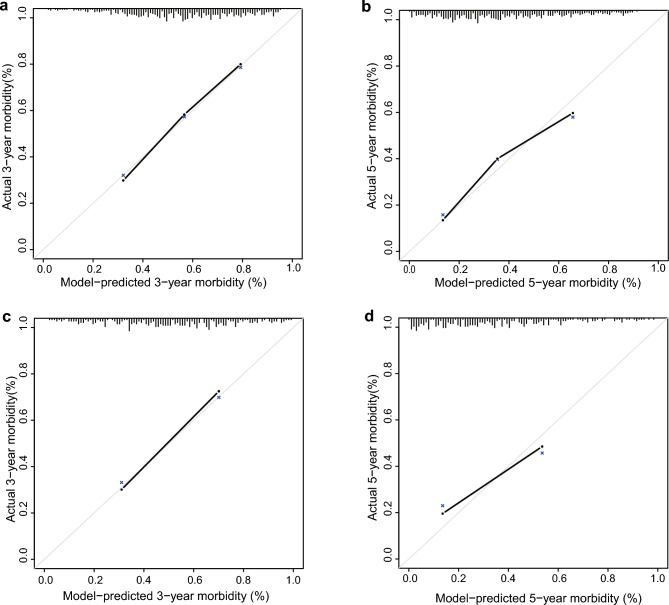
(**a**–**d**) 3-year and 5-year calibration curve of the model. The X-axis represents the model-predicted morbidity and the y-axis represents actual morbidity.

The calibration curves showed that the predictions of the cardiovascular event prediction model were in good agreement with the observations (Fig. 4a–d).

### Cross-validation of predictive models

Two methods, tenfold cross-validation and bootstrapping with 1000 resamples (70% derivation set and 30% validation set, repeated 1000 times) were used to validate the reliability of our model. The mean ROC for predicting the risk of cardiovascular events in MHDs at years 3 and 5 was 0.732 and 0.771, in tenfold cross-validation with 1000 resamples. In the bootstrapping with 1000 resamples, the derivation set Harrell’s c-index was 0.695 [95% CI, 0.677–0.714] and the validation set Harrell’s c-index was 0.667 [95% CI, 0.638–0.697] (eFigure 2 in the Supplement). Both validation methods demonstrated the predictive power of our cardiovascular event risk prediction model for MHDs.

## Discussion

We developed and validated for quantifying the association of cardiovascular events with the risk of 3- and 5-years for patients undergoing MHD. The study included 13 indicators (including age, mode of dialysis, whether or not intradialytic hypotension, waist-to-hip ratio, handgrip strength, extracellular water, lean tissue index, lymphocyte, platelet distribution density, total bilirubin, age on dialysis, and presence of hypertension and/or diabetes mellitus), all of which were evaluated and validated to confirm the reliability and accuracy of the model for predicting the risk of cardiovascular events in patients with MHD, providing a basis for cardioprotection in hemodialysis patients.

Protein-energy wasting (PEW) refers to a state of “malnutrition” in patients with chronic kidney disease in which various nutritional and metabolic abnormalities lead to a decrease in the body's protein energy reserves^6^. Studies have confirmed^7^ that during the development of PEW, a decrease in the rate of muscle protein synthesis and an increase in the rate of catabolism will cause progressive skeletal muscle wasting. Upper arm circumference, triceps skinfold thickness, calf circumference, body weight, and handgrip strength decreased with the progression of PEW, which seriously affected the quality of survival of patients^6^. This is consistent with our findings that the decrease in waist-to-hip ratio and handgrip strength with disease progression and dialysis treatment will increase the risk of cardiovascular events in patients. Several studies have shown that^8,9^ LTI correlates with skeletal muscle mass, reflecting the nutritional status of the patient, and that patients with low LTI have a significantly higher mortality rate than those with normal LTI, which is an independent predictor of mortality. Our results show that the loss of LTI is associated with the risk of cardiovascular events, which may be related to the decrease of fat reserve is more obvious than skeletal muscle.

Fluid overload is also one of the major risk factors for all-cause and cardiovascular mortality in MHDs, and we assess patient's nutritional and fluid loading status through the bioelectrical impedance. A recent study showed that^10^ patients with PEW and high ECW/ICW ratios were more likely to die from any cause and that the ECW/TBW ratio can be used to predict not only PEW, but also inflammation and volume overload in MHD, and is a strong predictor of mortality in patients with MHD.

The risk of cardiovascular disease-related death is increased approximately 30-fold in patients with end-stage renal disease receiving hemodialysis. Lipid oxidation or oxidative stress plays an important role in the pathogenesis of atherosclerosis. Previous studies have shown that serum bilirubin has potent antioxidant effects that are associated with the prevention of kidney injury and the reduction of cardiovascular events^11,12^. A large sample cohort study by Su et al. showed^13^ that higher TB levels were associated with higher mortality in MHDs without liver disease or abnormal liver function, and mildly elevated TB levels were not associated with a protective effect in MHDs. The authors’ explanation may be because total bilirubin is negatively correlated with nutrition and body mass index, and low body mass index is strongly associated with mortality in uremic patients.

Increased platelet activation and release will be presented as an increase in the density of platelet distribution Platelet. The activation and oxidative of platelets play a key role in atherosclerotic plaque instability and plaque rupture with subsequent thrombus formation in patients with ST-segment elevation myocardial infarction^14^. Platelet activation leading to the release of proinflammatory, pro-mitotic, and pro-apoptotic molecules and cytotoxic substances, as well as interactions with leukocytes and endothelial cells, will trigger the onset and amplification of myocardial ischemia–reperfusion injury^15^.

Microinflammation is the first step in the “inflammation-dystrophy-atherosclerosis” process in end-stage hemodialysis patients^16^, which is closely related to cardiovascular events. Our findings showed that lymphocyte counts were positively associated with the risk of cardiovascular events in maintenance hemodialysis patients, which may be related to the activation of the immune system by microinflammatory responses and the release of inflammatory mediators thereby impairing vascular endothelial function, ultimately leading to atherosclerosis and cardiovascular events. A retrospective study by Yanping Zhang et al. demonstrated^17^ that a high platelet-to-lymphocyte ratio (PLR) independently predicted all-cause mortality in patients with MHD and that a highly expressed PLR was associated with cardiovascular mortality.

Intradialytic hypotension (IDH) is associated with higher ultrafiltration volumes (roughly > 10–13 ml/h/kg in different studies)^18,19^, reduced cardiac output, and sluggish sympathetic activation. Based on IDH may lead to end-organ damage such as cerebral ischemia, mesenteric ischemia, accelerated loss of residual renal function, and is also associated with thrombosis and cardiac arrhythmias, which are independent risk factors for all-cause mortality^20–22^. Many studies have shown a “U-shaped” or “reverse J-shaped” relationship between blood pressure and mortality, suggesting that mortality is higher for lower systolic blood pressure (< 120 mmHg) pre- and post-dialysis (especially for those < 50 years of age and those with comorbid diabetes mellitus), and that higher systolic blood pressure (> 180 mmHg) is associated with slightly higher risk of death, and that the mortality due to low systolic blood pressure is attributable to cardiovascular complications^23^.

A prediction model for cardiovascular events in a Japanese hemodialysis population developed by Li et al. indicated^24^ that age, diabetes status, and frequency of dialysis were associated with the occurrence of cardiovascular events, which is consistent with our study. There is a positive correlation between the age of the patient and the risk of cardiovascular events. The decline in physical function with increasing age may lead to hardening of the blood vessel walls and a decline in cardiac function, resulting in an increased risk of cardiovascular events. However, the age of patients on dialysis was inversely associated with the risk of cardiovascular events. Studies have shown^25^ that mortality in HD patients ranges from 5.6–8.6% within 90 days of starting hemodialysis and 16.2–24.3% within one year, which may be related to poor renal function at the initial base of the disease, fluctuations in blood volume (alternating between hyper- and hypovolemia), activation of pro-inflammatory cytokines and complement, and nutritional status.

Compared to existing models, our clinical prediction model has many advantages. For example, in the research by Matsubara et al.^26^, the follow-up period for our model was much longer than theirs. It is critical that we have assessed the external validation of this model. And the sample size of our model is large enough compared to existing models, such as Li et al.^27^. It is important that the Model is concise, practical, and steady, and the predictors are intuitively derived from clinical work, which has high practicability. Nonetheless, there are limitations to this study, and the model requires external validation in a broader maintenance hemodialysis population.

In conclusion, we developed a model to estimate the cardiovascular events risk for patients undergoing MHD. We found that protein-energy expenditure plays a critical role in disease progression and prognosis in maintenance hemodialysis patients and is an important goal in delaying disease progression. Identifying potential pathophysiologic factors underlying cardiovascular events in maintenance hemodialysis patients is critical to reducing both the incidence of cardiovascular events and mortality in maintenance hemodialysis patients. All of the above relevant factors are associated with the risk of cardiovascular events in maintenance hemodialysis patients and could provide clinicians with appropriate references.

## Methods

### Data source and study population

This study enrolled 3488 MHDs from 20 hemodialysis centers in Guizhou Province from June 2015 to August 2020 and followed up until August 2021. We excluded MHDs (under 18 years old) (n = 676) that had cardiovascular events in the past, that had malignant tumors, and that had mental illness or severe aphasia so they could not cooperate with the questionnaire or total data missing (Greater than 20%). The final analytic cohort included the remaining 2812 MHD patients (eFigure 1 in the Supplement). This study followed the Transparent Reporting of a Multivariable Prediction Model for Individual Prognosis or Diagnosis (TRIPOD) reporting guidelines^28^. Patients refused to give written consent were excluded. Eligible participants provided informed consent. This study adhered to the Declaration of Helsinki. The study protocol was approved by the Clinical Research Ethics Committee of The Guizhou Provincial People’s Hospital (Ethical Word [2019] 29).

### Study outcome

The primary outcome was Major Adverse Cardiac and Cerebrovascular events (MACCE), defined by fatal/non-fatal myocardial infarction, heart failure, arrhythmia, and cerebrovascular disease. Nowadays, there is still a lack of medical consensus on the definition of intradialytic hypotension (IDH), and the differences may lead to an inability to accurately estimate IDH prevalence and outcomes. In recent years, the definitions provided in various guidelines include^29^, (i) any episode of a decrease in blood pressure (BP) during dialysis that requires immediate intervention, (ii) a symptomatic decrease in either systolic blood pressure (SBP) ≥ 20 mmHg or mean arterial pressure (MAP) ≥ 10 mmHg that needs intervention and (iii) a symptomatic sudden drop in SBP ≥ 30 mmHg or a decrease in MAP ≥ 10 mmHg. The final definition of IDH was selected for this study by the National Kidney Foundation’s Kidney Disease Outcomes Quality Initiative Guidelines in 2005, IHD is a decrease in either SBP ≥ 20 mmHg or MAP ≥ 10 mmHg leading to symptoms.

### Candidate variables

We used the physical measurement data and accessible laboratory indicators as risk factors to develop the model. Before the dialysis, the researcher used tape measures, weight scales, and sphygmomanometers to collect patient data, including height, pre-dialysis weight, waist circumference, hip circumference, calf circumference, blood pressure before, during, and after dialysis, and so on. Then a bioelectrical impedance analyzer (Germany, Fresenius BCM-7BJA4951) was used to make measurements, including intra- and extracellular water, somatic cell index, and so on. The professional researchers uniformly issue questionnaires and conduct one-to-one inquiries, including the patient’s gender, age, medical history, medication, and so on. Finally, we will access the medical record management system of each hospital to collect laboratory data such as routine blood counts, blood creatinine, and electrolytes from patients within the last 3 months.

### Statistical analyses

To derive and validate the model, we randomly divided the final analytic cohort into a derivation set (70% of the cohort) and internal and external validation set (respectively 20% and 10%). The model was built using the training set, and the performance of the model was evaluated on the internal and external validation.

### Missing data

Potential bias from missing data was evaluated by comparing the characteristics of patients with one or more missing predictor variables to patients with complete data. When more than 80% of the data was missing, it was directly deleted. Other missing data were assumed to be missing at random and imputed using multiple imputation techniques. Due to the large sample size, the proportion of outliers were treated as missing data and removed from the analyses.

### Model development and validation

First, univariate survival and multivariate survival Cox regression analyses were performed using R software. After univariate survival Cox regression analyses, the meaningful variables (*P* < 0.05) will be analyzed for PH assumption analyses through *Cox. zph* function (*P* > 0.05). And ensure that the risk is constant across individuals and the risk ratio is constant over time. These variables that met the PH assumption analyses were subjected to multivariate survival Cox regression analyses. We will perform Cross-LASSO regression analyses of survival data using the Glmnet R package. Cross-LASSO regression combines the methods of cross-validation and LASSO regression and can synthesize a balance of feature selection and model complexity. We performed multivariate survival Cox regression analyses based on cross-LASSO regression, which integrates the results of feature selection and adjusted variables to accurately estimate the hazard ratio. The final optimal set of parameters is determined by balancing the number of variables with the performance of the model [mainly based on the consistency index (Harrell’s C-index)].

The Cox proportional risk model was used to draw a visual Nomogram graph based on the optimized parameters. The predictive performance of the nomograms was evaluated by the area under the curve (AUC). The predictive accuracy and discriminatory power of the model were determined by the Harrell’s C-index and calibration plots. The Harrell’s C-index, which indicates the agreement between predicted and actual outcomes, was calibrated by comparing the mean of predicted and actual disease incidence rates. Calibration was performed using bootstrapping with 1000 research resamples and tenfold cross-validation (70% training set and 30% validation set, repeated 1000 times).

All analyses were developed in R version 4.3.0 (the R Foundation for Statistical Computing, version 4.3.0 https://www.r-project.org/). Continuous variables were presented as median [interquartile range (IQR)], and compared using the Mann–Whitney *U*-test. Categorical variables were summarized as frequencies and proportions (%) and compared using the χ^2^ test, as appropriate. *P* < 0.05 was considered statistically significant for tests. Multicollinearity between predictor variables was assessed by correlation coefficients and variance inflation factors, and we insisted that each variable had less than 10 degrees of freedom to minimize over-fitting. The LASSO regression was performed using the *survival*, *corrplot*, *glmnet*, and *foreign* R package. The PH assumption analyses were performed using the *Cox.zph* R package. The Cox proportional risk model and Harrell’s C-index were performed using the *RMS*, *survival*, *pec*, *Hmisc* R package. The ROC curve was performed using the *timeROC* package, the tenfold cross-validation was performed by *caret*, *riskRegression* R package and the bootstrapping with 1000 resamples was performed by the bootstrap R package.

### Supplementary Information

Supplementary Information.

## Data Availability

The datasets generated and/or analysed during the current study are not publicly available due to protect the privacy of the individuals included in the study but are available from the corresponding author on reasonable request.
