# Supplementary material for: Development and validation of a model for predicting the risk of cardiovascular events in maintenance hemodialysis patients
Source: Sci Rep. 2024 Mar 21;14:6760. doi: 10.1038/s41598-024-55161-y (PMC10958022; doi:10.1038/s41598-024-55161-y)
Supplement: Supplementary file 1 — Supplementary Information. [file 41598_2024_55161_MOESM1_ESM.docx]

**Supplementary Content**

**eFigure 1.** Study Flow for Model Development and Internal and Temporal Validations

**eFigure 2.** The bootstrapping with 1000 resamples of derivation and validation

**Table 1.** Baseline characteristics of the study populations

**eTable 2.** Univariate survival COX regression analysis

**eTable3.** Multivariate survival COX regression analysis

**eFigure 1. Study Flow for Model Development and Internal and Temporal Validations**


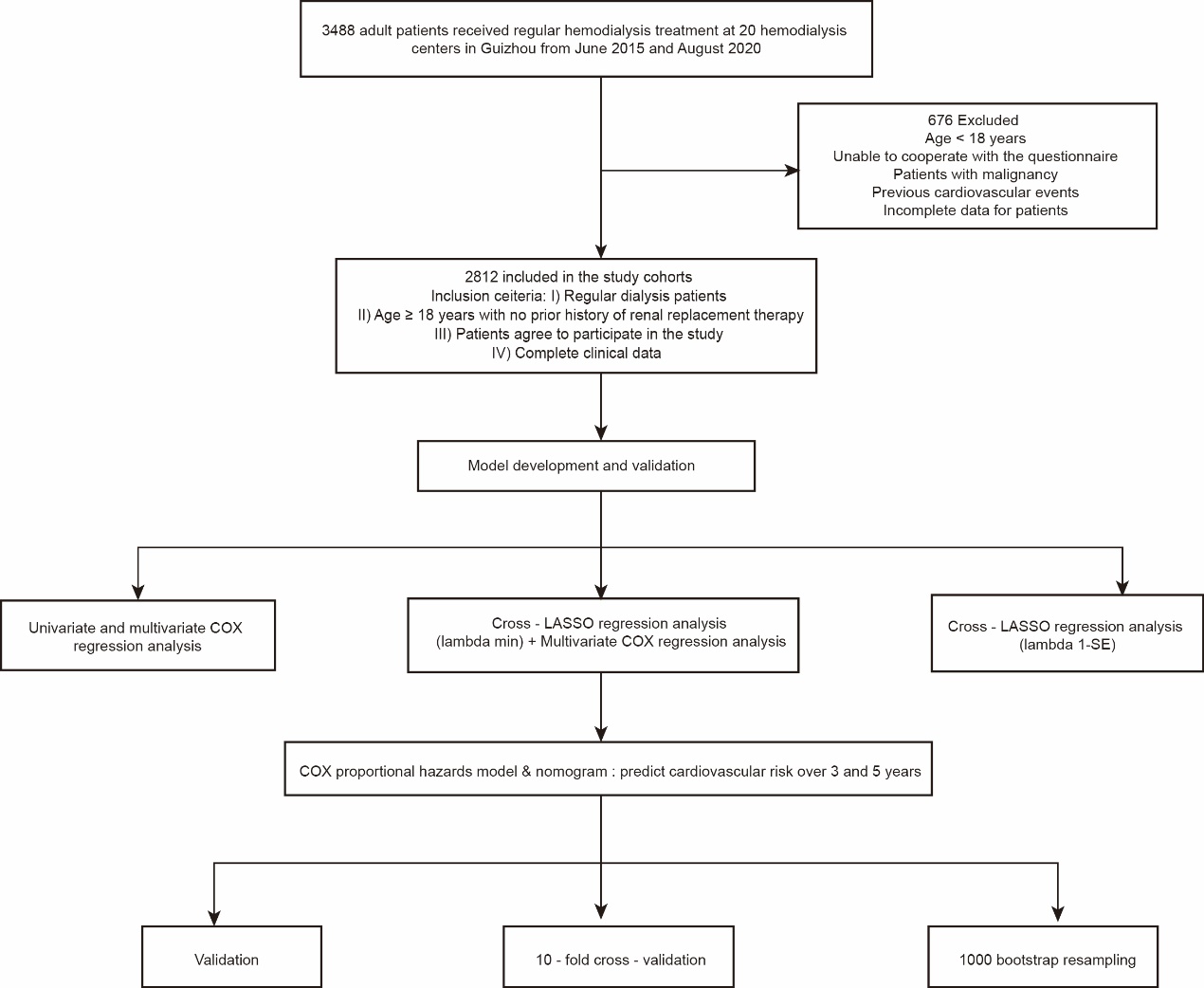


**eFigure 2. The bootstrapping with 1000 resamples of derivation and validation**


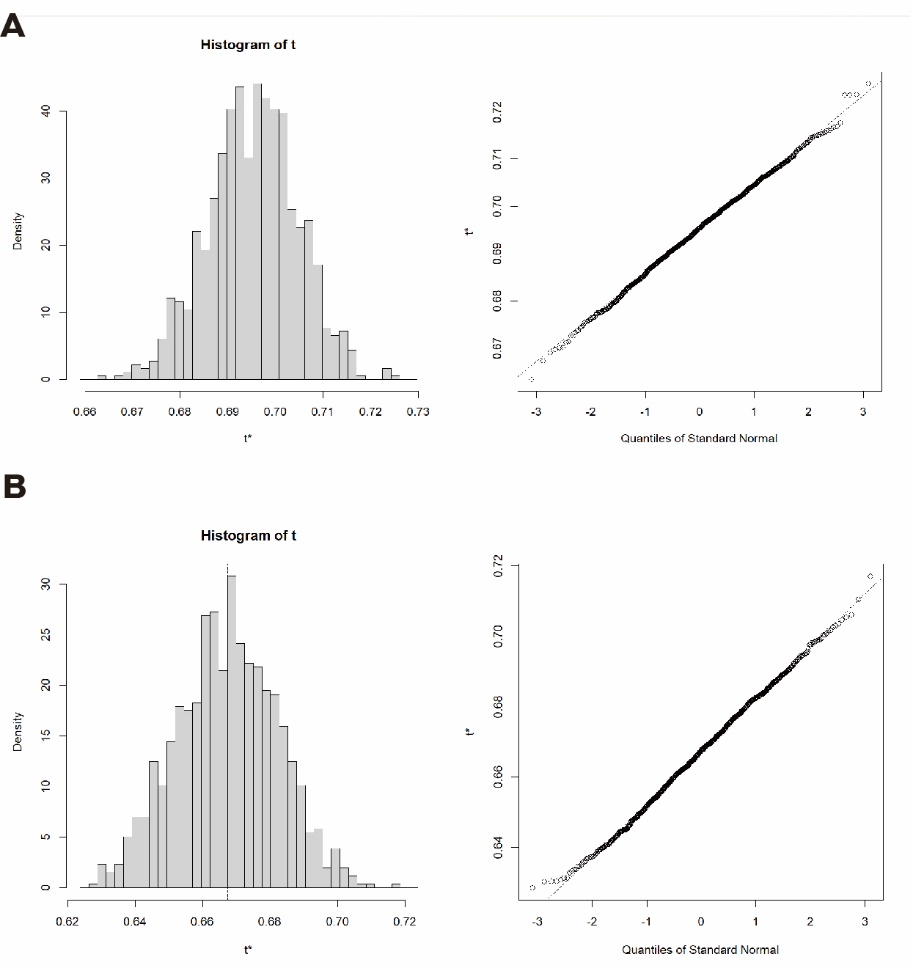


In the bootstrapping with 1000 resamples, the derivation set c-index was 0.695 [95% CI:0.677-0.714] and the validation set c-index was 0.667 [95% CI:0.638-0.697].

**Table 1.** **Baseline characteristics of the study populations**

|  | cardiovascular event | No-cardiovascular event |
| --- | --- | --- |
| Sex |  |  |
| Man | 939(59.1%) | 755(61.7%) |
| female | 649(40.9%) | 469(38.3%) |
| Dialysis mode (HD/ HD+HP; HDF) |  |  |
| HD/ HD+HP | 1058(66.6%) | 812(66.3%) |
| HDF | 530(33.4%) | 412(33.7%) |
| Intradialytic hypotension (yes/no) |  |  |
| 1 | 426(26.8%) | 194(15.8%) |
| 2 | 1162(73.2%) | 1030(84.2%) |
| Intradialytic hypertension (yes/no) |  |  |
| 1 | 530(33.4%) | 381(31.1%) |
| 2 | 1058(66.6%) | 843(68.9%) |
| Dialysis frequency |  |  |
| 1 | 14(0.9%) | 13(1.1%) |
| 2 | 239(15.1%) | 219(17.9%) |
| 3 | 1325(83.4%) | 989(80.8%) |
| 4 | 10(0.6%) | 3(0.2%) |
| Hemofiltration frequency |  |  |
| 0 | 252(15.9%) | 268(21.9%) |
| 1 | 241(15.2%) | 314(25.7%) |
| 2 | 618(38.9%) | 304(24.8%) |
| 3 | 252(15.8) | 153(12.5%) |
| 4 | 225(14.2%) | 185(15.1%) |
| Hemoperfusion frequency |  |  |
| 0 | 336(21.2%) | 331(27.0%) |
| 1 | 29(1.8%) | 38(3.1%) |
| 2 | 158(9.9%) | 103(8.4%) |
| 3 | 346(21.8%) | 183(15.0%) |
| 4 | 719(45.3%) | 569(46.5%) |
| Hypertension (yes/no) |  |  |
| 1 | 1481(93.3%) | 957(78.2%) |
| 2 | 107(6.7%) | 267(21.8%) |
| Diabetes (yes/no) |  |  |
| 1 | 575(36.2%) | 266(21.7%) |
| 2 | 1013(63.8%) | 958(78.3%) |

**eTable 2 Univariate survival COX regression analysis**

|  | HR | P |
| --- | --- | --- |
| Age | 1.009(1.005-1.013) | ＜0.000 |
| Dialysis mode (HD/ HD+HP; HDF) | 0.874(0.772-0.989) | 0.034 |
| Intradialytic hypotension (mmHg) | 1.363(1.194-1.556) | ＜0.000 |
| WHR (cm) | 0.242(0.109-0.536) | 0.000 |
| Handgrip (kg) | 0.989(0.983-0.996) | 0.002 |
| ECW | 1.034(1.015-1.054) | 0.000 |
| LTI | 1.027(1.008-1.045) | 0.005 |
| HB g/L) | 0.997(0.994-0.999) | 0.024 |
| NEUT *10^9^/L) | 1.034(1.002-1.067) | 0.037 |
| LYMPH *10^9^/L) | 1.094(1.032-1.160) | 0.003 |
| MONO *10^9^/L) | 1.299(1.189-1.419) | ＜0.000 |
| Plt density fL) | 1.022(1.010-1.033) | 0.000 |
| TB μmol/L) | 1.013(1.001-1.024) | 0.031 |
| UA μmol/L) | 0.9992(0.998-0.9997) | 0.002 |
| Dialysis vintage year) | 0.881(0.862-0.901) | ＜0.000 |
| Hypertension yes/no) | 2.186(1.747-2.732) | ＜0.000 |
| DM yes/no) | 1.568(1.389-1.771) | ＜0.000 |

Abbreviation: ECW, Extracellular water; HB, Haemoglobin; HD, Hemodialysis; HP, Hemoperfusion; HDF, Hemodiafiltration; LTI, Lean tissue index; LYMPH, Number of lymphocytes; MONO, Monocyte count; NEUT, Proportion of neutrophils; TB, Total bilirubin; UA, Uric acid; WHR, Waist-hip ratio

univariate survival COX regression analysis was performed using R software to elect 17 predictors.

**eTable 3 Multivariate survival COX regression analysis**

|  | HR | P |
| --- | --- | --- |
| Age | 1.005(1.003-1.230) | 0.04 |
| Dialysis mode | 0.867(0.765-0.983) | 0.026 |
| Intradialytic hypotension (mmHg) | 1.308(1.144-1.496) | ＜0.000 |
| WHR (cm) | 0.144(0.764-0.897) | ＜0.000 |
| Handgrip (kg) | 0.988(0.786-0.945) | 0.002 |
| LTI | 1.026(1.009-1.209) | 0.032 |
| LYMPH (*10^9^/L) | 1.096(1.017-1.089) | 0.032 |
| Plt density (fL) | 1.014(1.0144-1.223) | 0.012 |
| TB (μmol/ L) | 1.017(1.0223-1.147) | 0.006 |
| Dialysis vintage (year) | 0.889(0.564-0.677) | ＜0.000 |
| Hypertension (yes/no) | 1.933(1.540-2.426) | ＜0.000 |
| DM (yes/ no) | 1.308(1.141-1.499) | 0.000 |

Abbreviation: LTI, Lean tissue index; LYMPH, Number of lymphocytes; TB, Total bilirubin; UA, Uric acid; WHR, Waist-hip ratio

**eTable 4 Model 2**

|  | HR | P |
| --- | --- | --- |
| Age | 1.005(1.007-1.237) | 0.037* |
| Dialysis mode | 0.870(0.767-0.987) | 0.030* |
| Intradialytic hypotension (mmHg) | 1.372(1.182-1.592) | ＜0.000* |
| WHR (cm) | 0.149(0.767-0.900) | ＜0.000* |
| Handgrip (kg) | 0.989(0.804-0.977) | 0.015* |
| LTI | 1.024(1.002-1.201) | 0.046* |
| LYMPH (*10^9^/L) | 1.091(1.015-1.086) | 0.005* |
| Plt density (fL) | 1.014(1.013-1.122) | 0.014* |
| TB (μmol/L) | 1.016(1.006-1.158) | 0.033* |
| Dialysis vintage (year) | 0.888(0.563-0.676) | ＜0.000* |
| Hypertension (yes/no) | 1.878(1.494-2.359) | ＜0.000* |
| DM (yes/no) | 1.313(1.143-1.508) | 0.000* |

Abbreviation: LTI, Lean tissue index; LYMPH, Number of lymphocytes; TB, Total bilirubin; UA, Uric acid; WHR, Waist-hip ratio
